# Lessons Learned and Future Actions: Modifying a Stroke Specific Self-Management Program

**DOI:** 10.3389/frhs.2022.841082

**Published:** 2022-06-21

**Authors:** Kimberly Hreha, Mandi Sonnenfeld, Annalisa Na, Riqiea Kitchens, Timothy A. Reistetter

**Affiliations:** ^1^Division of Occupational Therapy, Department of Orthopaedic Surgery, School of Medicine, Duke University, Durham, NC, United States; ^2^University of Texas Medical Branch, Division of Rehabilitation Science, Galveston, TX, United States; ^3^Physical Therapy and Rehabilitation Sciences Department, Drexel University, Philadelphia, PA, United States; ^4^University of Texas Medical Branch, Department of Occupational Therapy, Galveston, TX, United States; ^5^Department of Occupational Therapy University of Texas Health Science Center at San Antonio, San Antonio, TX, United States

**Keywords:** stroke, implementation science, consolidated framework for implementation research, self-management, translational research

## Abstract

**Background:**

Self-management programs have been shown to be effective at providing support to individuals who want to manage chronic health conditions independently. It has been shown that adapting self-management programs for different diagnostic groups, such as stroke, is essential.

**Objective:**

To report modifications made during trial implementation, the barriers identified during the delivery of an evidence based, stroke-specific self-management program and minor data (including strategies made) from a small cohort of stroke survivors with multiple chronic conditions.

**Methods:**

Prospective type III hybrid implementation-effectiveness trial for stroke survivors, with chronic conditions, living in the community, and interested in self-management. Modifications were reported by the following: (1) researcher reflections (2) barriers to implementation and (3) strategies used to address the barrier using the Consolidated Framework for Implementation Research (CFIR) guidelines from field notes.

**Results:**

Twenty-five individuals consented (42% of eligible sample) at the time of acute stroke and five were interested in continuing at the 3-month call. Multiple barriers to implementation were identified, resulting in modifications. For example, before the group sessions began, the COVID-19 pandemic necessitated changes to the intervention delivery. The protocol was modified to an online mode of delivery. In total, there were seven modifications made.

**Conclusions:**

The CFIR was a facilitative tool to report barriers and strategies and emphasized the importance of comprehensive reporting. The modifications to the study were an essential first step to address the research climate and needs of this stroke cohort. Next steps include continued research with a larger cohort to implement effective strategies and answer the clinical question of effectiveness of the adapted and modified intervention.

## Introduction

Despite comprehensive rehabilitation programs and supportive care, many individuals who have sustained a stroke cannot effectively manage residual stroke symptoms in addition to existing comorbidities in order to live independently at home and therefore must develop strategies to gain new knowledge, skills and confidence (1). In addition, lack of access to interventions and variable quality of care at different points in the post-stroke pathway are issues that prevent improvement (2). Self-management programs are effective at supporting and empowering individuals with chronic conditions (such as stroke), by teaching the skills necessary to actively and independently manage symptoms (1).

Many self-management programs have been developed and are being used by multiple patient populations. One example is the Chronic Disease Self-Management Program (CDSMP), an evidence-based self-management program that has been shown to be effective at improving overall health, health service utilization, and self-efficacy of individuals participating in the program (3, 4). The CDSMP curriculum has been adapted (prior to it being delivered) and modified (during delivery) for stroke survivors and used at multiple stages of stroke recovery (5, 6). These two studies demonstrated feasibility and improvements such as self-efficacy in the stroke group vs. the group that did not receive the intervention (5, 6). Another program added education on home, community, and work management, and yielded effective improvements in self-efficacy for health behavior management and participation (7). It is unknown whether further specific modification and tailoring of the program that not only focuses on the stroke symptoms but also on the coexisting health diseases that each person is experiencing will improve outcomes. Since most stroke survivors have multiple chronic conditions (8), specifically adapting the CDSMP to meet the needs of this cohort is a gap.

In 2019, we made adaptations to the CDSMP, using a visual analytic methodology and using medical records of stroke survivors with chronic conditions (9). These adaptions also included the development of clinical vignettes which were intended to be used to create tailored discussion opportunities and more personalized content for CDSMP future participants (9). The clinical vignettes relate to the weekly sessions' content and are situated within the curriculum during scheduled discussions and therefore keep the CDSMP fidelity (9).

After the adaptations were made, we intended to then conduct a type III hybrid implementation-effectiveness study to make any modifications as well as evaluate the impact of the adapted self-management program, assessing both clinical and implementation outcomes. The purpose being to expedite the translation of research findings into clinical practice by generating more effective implementation strategies and information for decision makers. Therefore, this report describes modifications made during trial implementation, barriers identified during the delivery of an evidence-based stroke-specific self-management program and presents minor data (including strategies made) from five participants.

## Methods

### Study Design and Procedures

After full review, the stroke-specific CDSMP type III hybrid implementation-effectiveness (10) study was approved by the Institutional Review Board (IRB) at the University of Texas Medical Branch. Recruitment took place in the acute care hospital from August 2019 through February 2021 Medical records were used prospectively to screen new admissions and determine if inclusion criteria were met. Patients were approached to determine their interest in the study after discussion with their nurse. Consent and baseline 1 assessments were completed in participants' hospital rooms by the principle investigator (researcher). The process took ~45 min. The assessment testing was repeated at two additional time points during the study (prior to the intervention and 2 weeks after). These assessments include multiple clinical outcome measures and are not reported in this manuscript. They are: (1) Southampton Stroke Self-Management Questionnaire (11), (2) Patient Reported Outcome Measure (PROMIS) self-efficacy (12), (3) PROMIS sleep disturbance scale (13), (4) PROMIS sleep-related impairment scale (13) and (4) visual functioning questionnaire (14).

**Figure 1:**
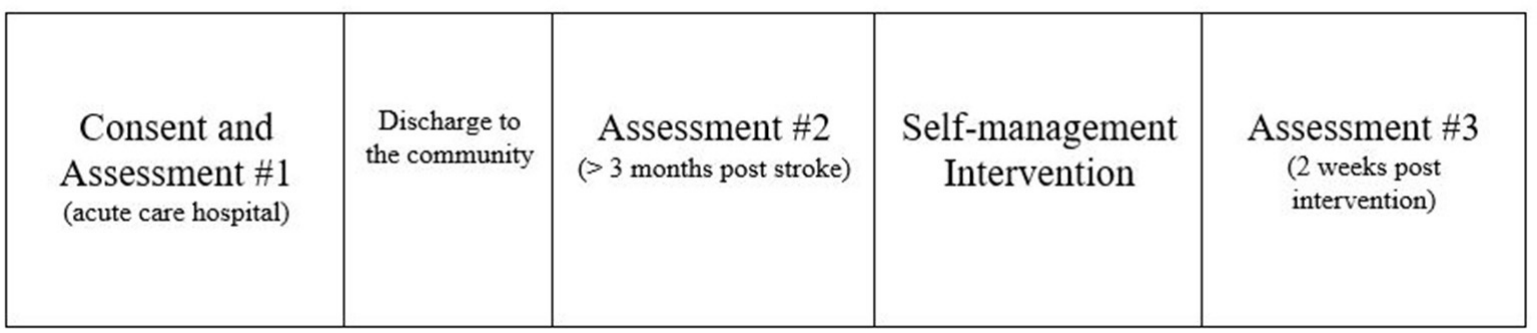
Schematic of study design.

Approximately 3 months after the consent and baseline 1 were complete, each person received a telephone call (see Appendix 1 for telephone script) from the study staff to complete a brief interview. This interview determined if the person still met the study's inclusion criteria, asked if they were interested in continuing the study, provided a timeline for when the second assessments needed to be completed, and identified the person's optimal day and time for when they could participate in the weekly group sessions. The study staff (occupational therapist) provided the following additional information during the call: information on the specific location for in-person sessions, parking information, including how to be compensated, and a reminder that family members were welcome to attend the group sessions. An honorarium was provided after the second set of assessments was completed. The study investigators and staff were trained in the CDSMP as group facilitators prior to the study being implemented. Over a 6-week period, the principle investigator and study staff lead the group members through the implementation of the intervention. The final assessments were completed after the last intervention group meeting (see Figure 1).

The principle investigator and study staff (researchers) took field notes before and during the intervention. Barriers to implementation were reported using the Consolidated Framework for Implementation Research (CFIR) which was done after the intervention through utilization of field notes. Researcher reflections were used to make modifications to the study protocol.

### Participants

Participants for this study were required to meet the following inclusion criteria: diagnosis of an acute stroke, living with at least one chronic condition able to independently consent (in other words, each person was alert and oriented to person, place, and date), be community dwelling at the start of the intervention, and be over the age of 18 years. The chronic disease definition used to determine inclusion was: a medical condition that persisted more than 1 year and either requires ongoing medical attention and/or results in limitations in activities of daily living (15).

### Intervention

The intervention for this study was the six-week CDSMP workshop, originally developed by Lorig et al. (4) and led by two trained CDSMP facilitators. The group sessions included six learning modules, one for each week of the workshop. Examples of the topics discussed were exercise, symptom management, nutrition, sleep and fatigue management, emotion management, communication training, health-related problem solving, and decision-making (4). This information was also published in the CDSMPBook, which was given to each participant to aid intervention delivery (16). The sessions were completed in group format one time per week and lasted ~2 h.

### Data Collection and Analysis

The principle investigator monitored the number of patients screened, eligible, approached, and enrolled as well as any study refusals, withdrawals, lost-to-follow-up, and adverse events. We used REDCap software system to obtain and store data, including demographics and assessment results. As explained above, the telephone questionnaire was used to determine personal reasons why consented participants were or were not interested in continuing the study. This information was kept in a password protected Excel file. This file was also used to collect any researcher field notes, which included comments noted verbally by patients during the intervention group sessions, and personal reflections.

The CFIR framework was incorporated to systematically define barriers as well as report strategies used to attempt to eliminate the identified barriers. The CFIR is comprised of five domains, which include: intervention characteristics, outer setting, inner setting, characteristics of the individuals involved and the process of implementation (17). In addition, a total of 37 constructs related to the domains are indicated as either a facilitator or a barrier (17). For example, intervention characteristics is the first domain and includes constructs such as intervention source, adaptability and cost (17).

## Results

A total of 352 patient medical charts were screened. Fifty-nine individuals met the inclusion criteria. Despite meeting the inclusion criteria, 18 individuals were discharged from the hospital before being approached and 16 individuals declined participation at initial approach. Consent and assessments were completed for 25 people (42% of the eligible sample). Out of the 25 patients, five (20%) indicated an interest in continuing the study, completed the second assessment battery and were scheduled to participate in the six intervention sessions. There were 11 withdrawals, of which was one of the five that indicated interest after the second assessment was completed, and 10 lost-to-follow-up. The demographics and characteristics of these five participants are reported in Table 1.

**Table 1:** Five trial participants' demographics and characteristics.

**Characteristic**	**Frequency**
Male, *N* (%)	2 (40%)
Age, mean (SD)	58.96 (2.45)
Non-Hispanic ethnicity	5 (100%)
**Race**, ***N*** **(%)**
Black or African American	2 (40%)
White	3 (60%)
Acute hospital length of stay, days (SD)	7 (2.45)
**Discharge destination after acute care stay, N (%)**
Inpatient rehabilitation	1 (20%)
Home	3 (60%)
Skilled nursing facility	1 (20%)
**Stroke location, N (%)**
Left stroke	3 (60%)
Right stroke	2 (40%)
**Comorbidities, N (%)**
Previous stroke	1 (20%)
Hypertension	4 (80%)
Diabetes	2 (40%)
Hyperlipidemia	3 (60%)
Tobacco abuse	1 (20%)
Chronic obstructive pulmonary disease	1 (20%)
Mental illness	1 (20%)
Other	4 (80%)
**Vision conditions, N (%)**
Glaucoma	1 (20%)
Cataract	1 (20%)
Visual field cut	1 (20%)
Visual acuity impairment	1 (20%)
Other vision impairment	3 (60%)

*N, number; %, percentage; SD, standard deviation*.

All participants completed the first session, however did not attend session two, even after study staff attempted to engage these participants in multiple ways (e.g., email, phone calls). Because the intervention was designed to be delivered in a group format, we paused the study to determine next steps. However, it is important to note that, even before this outcome occurred, the study staff identified multiple barriers and attempted to determine strategies to address these barriers during the implementation phase. Table 2 describes the barriers encountered using the CFIR framework and reports the attempted strategies used to remove each barrier. For example, for the construct “External Policies and Incentives” that is noted in “Domain II: Outer Setting” (Table 2), the related barrier is the COVID-19 pandemic and the University mandate to suspend all in-person research. In response, we modified the in-person protocol to a format that can be implemented via a virtual platform, Zoom, a HIPAA compliant telehealth-based technology. This modification required a protocol amendment that was approved by the University's Review Board. There were seven modifications made in total during the implementation up to the time when the study was paused (see Table 2).

**Table 2:** Barrier assessment by domains of the consolidated framework for implementation research (CFIR) with modifications to remove the barrier.

**Construct**	**Barrier**	**Strategy/modification to remove the barrier**
**Domain I: Intervention characteristics**		
Adaptability	• Virtual technology was not available to participants: ° Did not have computers or tablets ° Did not have internet	• Phone option: ° We suggested using phones and a conference call line (modification # 1) • Study staff either traveled to the participant's homes to provide the study materials or mailed information needed for participation (modification # 2)
Complexity	• Phone option: ° Difficult for participants to follow the content because the workshop online use/following PowerPoint slides and when it was in-person, whiteboards and posters were used ° Also, the workshop encourages participant engagement activities such as pairing off into smaller groups for discussions	We printed out all materials so that participants would be able to follow when on the phone (modification # 3)
Cost	• We did not anticipate the study changing into the virtual format; therefore, we did not purchase iPads for each participant and therefore had to resort to the phone • Also, when we changed to the virtual format, we did not anticipate that internet was not accessible to everyone	• Study investigator reapplied for funding
**Domain II: Outer setting**		
Patient needs and resources	• The COVID-19 pandemic affected patients' needs and resources because everything was shut down and then then eventually required new protocols to be followed • The phone method did not appear to meet patients' needs	• We called and informed participants that all aspects of research are postponed until further notice • We notified them that they would need to re-sign the consent form that has been modified to allow virtual participation • We ended up postponing the group due to low participation
Cosmopolitanism	• The evidence-based practice intervention being implemented in this study was designed by the Stanford Chronic Disease Self-Management group. We are required to follow the protocols they set, which is an in-person, over 6 member group	• One of the facilitators attended webinars hosted by Lorig et al., which was developed to roll out the virtual format that is required to be followed by all trained group facilitators. The materials (PowerPoint) were shared online (modification # 4)
External policies and incentives	• COVID-19 pandemic led to suspending all in-person research	• We stopped in-person research and then revised our Institutional Review Board (IRB) documents to use a virtual platform, and deliver the intervention via tele-health, in order to continue the study (modification # 5) • We used REDCap to virtually complete all assessments with participants (modification # 6)
**Domain III: Inner setting**		
Networks and communications	• All communication was done through leadership and then via email, which could easily not be shared or was missed because there were so many new policies and procedures related to the pandemic each day	• Study staff were required to pay close attention to all news briefs being put out by the University in order to determine when they could start revising and submit the IRB • Study staff would check in with leadership routinely to determine if any communication was missed or if there were new rules to follow
**Implementation climate**		
Compatibility	• Change in mode of communication was initially difficult because all research materials were on the University Campus • Recruitment took place on campus at the hospital	• We used REDCap to access patient information securely until we could return to campus • We followed all hospital protocols, including obtaining personally fitted N95 masks before returning to the hospital floor
Knowledge and beliefs about the intervention	• The participants did not have any knowledge about the self-management program, even after sharing information during the consent process. A few decided not to continue with the group because they did not think it would be helpful to them	• We started to provided more information about the program through printed materials as well as verbally (modification # 7)
**Domain V: Process**		
**Construct**	**Barrier**	**Strategy/modification used to remove the barrier**
Planning	• Time involved in planning all aspects of the study • Time to get the IRB approved	• Lists, strategizing, participating in webinars • Completing the IRB approval process as early as possible

In addition, the researchers field notes summarized that participants did not participate after the first session for one to two reasons. Three participants disliked using the conference call line because they could not hear the other participants well. Four participants had difficulty with following the course content on the phone using the paper copy of the PowerPoint presentation. A researcher reflection included that the pandemic was occurring at the same time this intervention was attempting to be carried out and participants appeared to be overwhelmed.

## Discussion

This brief report seeks to discuss modifications made during trial implementation, the barriers identified during the delivery of an evidence based, stroke-specific self-management program and minor data (including strategies made) from five participants.

The CFIR provided structure to report barriers and specific strategies and/or modifications developed to remove the barriers. This method of reporting is not new and found to be effective in a clinical research environment (17). Also our findings were similar to another study that also found virtual efforts affecting clinical research activities and outcomes (18). Here we identified barriers such as the patients' lack of access to materials needed for telehealth. Another barrier was cost. We had purchased all materials necessary to complete the in-person workshops, but not for a virtual format. In addition to the participants in the group needing technology, including internet, we also needed a budget to deliver intervention materials to the participants because the CDSMP book was continued to be endorsed as a necessary material to be used even in the virtual environment.

Out of the seven modifications, there were two that were required and instructed by the original CDSMP team in order to maintain fidelity, as the transition to remote activities was not occurring just at our institution but worldwide (18). For example, we were required to use a PowerPoint presentation as the alternative for the physical white board charts that should be used when in the classroom environment.

There were a few lessons learned during the process of addressing barriers. For example, we attempted to use a phone option to address technology barriers, however, we did not determine prior to the modification if this was an appropriate strategy for all group members. The barrier that resulted in response to this modification was that the intervention had to pause because participants could not complete the activities required of the CDSMP. Also, a research reflection was that it was difficult to not be able to see participants' and any non-verbal gestures they might be making. Therefore, engagement and group participation became difficult. Another lesson learned was that we should have asked the participants, in real time, their opinion about the strategy being used. For example, was it appropriate? This might have helped determine new ideas or different actions to take rather than having to pause the intervention due to lack of participation.

In conclusion, this study contributes to the literature by increasing the understanding of barriers, modifications used and lessons learned, as we navigated the initiation of a type III hybrid implementation-effectiveness trial for individuals with stroke and chronic comorbidities. Telemedicine, while it can potentially overcome geographic and transportation barriers (18) that are common for people with conditions such as stroke, could bring on barriers or additional challenges, as we experienced in this study. We plan to resume the study with a new cohort, to evaluate the CSDMP program, implement strategies to the lessons we learned, as well as report clinical and implementation outcomes.

## Data Availability Statement

The datasets presented in this article are not readily available because of patient confidentiality. Requests to access the datasets should be directed to the corresponding author.

## Ethics Statement

The studies involving human participants were reviewed and approved by University of Texas Medical Branch. The patients/participants provided their written informed consent to participate in this study.

## Author Contributions

KH obtained IRB approval, and ran the study, collected data, and wrote the manuscript. AN analyzed the data. RK assisted with study implementation. MS assisted with all aspects of the paper. TR determined the study design, helped with IRB approval, and wrote/edited the paper. All authors contributed to the article and approved the submitted version.

## Funding

This work was supported by: National Institutes of Health (UL1TR001439, K12 HD055929).

## Conflict of Interest

The authors declare that the research was conducted in the absence of any commercial or financial relationships that could be construed as a potential conflict of interest.

## Publisher's Note

All claims expressed in this article are solely those of the authors and do not necessarily represent those of their affiliated organizations, or those of the publisher, the editors and the reviewers. Any product that may be evaluated in this article, or claim that may be made by its manufacturer, is not guaranteed or endorsed by the publisher.
